# Benefits of D-005, a lipid extract from *Acrocomia crispa* fruits, in the prevention of acute kidney njury induced by nephrotoxicity in rats

**DOI:** 10.1590/2175-8239-JBN-2021-0048

**Published:** 2021-07-21

**Authors:** Sandra Rodríguez-Salgueiro, Leyanis Ocaña-Nápoles, Ambar Oyarzábal-Yera, Lucía González-Núñez, Giselle Breña-Betancourt, María Flavia Pérez-Pino, José A. Medina-Pírez, Sonia Jiménez-Despaigne, Vivian Molina-Cuevas

**Affiliations:** 1 Centro Nacional de Investigación Científica, Departamento de Farmacología, Havana, Cuba.; 2 Facultad Latinoamericana de Medicina, Departamento de Ciencias Morfológicas, Havana, Cuba.

**Keywords:** Acrocomia crispa, Renal Toxicity, Aminoglycoside Antibiotics, Oxidative Stress, Inflammation, Acrocomia crispa, Toxicidade, Renal, Antibióticos Aminoglicosídeos, Estresse oxidativo, Inflamação

## Abstract

**Introduction:**

Aminoglycoside-induced acute kidney injury (AKI) is a pathology closely linked to oxidative and inflammatory reactions. Taking into account the previous reported antioxidant and anti-inflammatory effects of D-005, a lipid extract obtained from Cuban palm *Acrocomia crispa* (Arecaceae) fruits, this work aimed to evaluate the effects of D-005 on kanamycin-induced AKI.

**Methods:**

Male Wistar rats were divided into 7 groups: negative control (vehicle, Tween 65/H2O) and six groups treated with kanamycin to induce AKI: positive control (vehicle), D-005 (25, 100, 200, and 400 mg/kg) and grape seed extract (GSE, 200 mg/kg). D-005, vehicle, and GSE oral treatments were administered once daily for seven days, 1 h before kanamycin (500 mg/kg, i.p.). Serum uric acid and urea concentrations, renal histopathology, and oxidative markers (malondialdehyde (MDA), sulfhydryl (SH) groups, and catalase (CAT) activity) were assessed.

**Results:**

D-005 significantly reduced uric acid and urea levels, starting from D-005 100 mg/kg. Histopathologically, D-005, at all the tested doses, protected renal parenchyma structures (glomeruli, proximal tubules, and interstitium). These findings were accompanied by a significant reduction of MDA and SH group concentrations as well as restoration of CAT activity. The highest percentages of inhibition were obtained with the dose of 400 mg/kg. GSE, the reference substance, also prevented kanamycin-induced biochemical and histopathological changes, as well as reduced MDA and SH groups and restored CAT activity.

**Conclusion:**

The administration of repeated oral doses of D-005 significantly protected against kanamycin-induced AKI, which could be associated with the antioxidant and anti-inflammatory effects of this extract.

## Resumo

**Introdução:**

Lesão renal aguda induzida por aminoglicosídeos é uma patologia intimamente ligada a reações oxidativas e inflamatórias. Considerando efeitos antioxidantes e anti-inflamatórios relatados anteriormente do D-005, um extrato lipídico de frutos da palmeira cubana *Acrocomia crispa* (Arecaceae), este trabalho avaliou efeitos do D-005 na LRA induzida por canamicina.

**Métodos:**

Dividiu-se ratos Wistar machos em 7 grupos: controle negativo (veículo, Tween 65/H2O) e seis grupos tratados com canamicina para induzir LRA: controle positivo (veículo), D-005 (25, 100, 200, 400 mg/kg) e extrato de semente de uva (ESU, 200 mg/kg). D-005, veículo, e tratamentos orais com ESU foram administrados uma vez por dia durante sete dias, 1 h antes da canamicina (500 mg/kg, i.p.). Avaliou-se concentrações séricas de ácido úrico e ureia, histopatologia renal e marcadores oxidativos (malondialdeído (MDA), grupos sulfidrila (SH), atividade de catalase (CAT)).

**Resultados:**

D-005 reduziu significativamente níveis de ácido úrico e ureia, partindo de D-005 100 mg/kg. Histopatologicamente, D-005, em todas as doses testadas, protegeu estruturas do parênquima renal (glomérulos, túbulos proximais e interstício). Estes achados foram acompanhados por uma redução significativa das concentrações de MDA e grupo SH, e pela restauração da atividade CAT. As maiores porcentagens de inibição foram obtidas com a dose de 400 mg/kg. ESU, a substância de referência, também evitou alterações bioquímicas e histopatológicas induzidas por canamicina, reduziu MDA e grupos SH e restaurou atividade CAT.

**Conclusão:**

A administração de doses orais repetidas de D-005 protegeu significativamente contra LRA induzida por canamicina, que pode estar associada aos efeitos antioxidantes e anti-inflamatórios deste extrato.

## Introduction

Acute kidney injury (AKI) is a clinical syndrome of varied etiology characterized by a sudden decrease in kidney function, accumulation of waste metabolites, water-mineral imbalance, and systemic symptoms. It is a common problem in intensive care medicine since a moderate AKI increases the risk of death by approximately five times^1^^,^ 2.

Several drugs usually used in the clinical setting, including aminoglycoside antibiotics (AMGs), contribute to AKI due to their nephrotoxic side effects. AKI induced by AMG treatment has a worldwide incidence of 5 to 15 %, appearing 7 to 10 days after starting the administration of the AMG. The immediate clinical manifestations are: oliguria with dysfunction of renal excretion and increased serum creatinine, urea, and other metabolic products^3^.

The accumulation of AMG in the tubular epithelium triggers the initial phase of AKI leading to a complex interrelation of vascular and tubular processes causing kidney dysfunction. Tubular toxicity by AMG is mainly characterized by several pathological changes in tubular epithelial cells in the proximal segment, associated with an important inflammatory component followed by non-lethal functional alterations of key components involved in the transport of water and solutes^4^^,^ 5.

As a consequence of AMG-induced tubular cell damage, some epithelial cells detach leading to formation of casts, composed of cells and proteins, into tubular lumens. The casts obstruct tubular lumens, causing an increase in intratubular pressure, which reduces the excretory function of affected nephrons and elevates the hydrostatic pressure in the Bowman's capsule, thus decreasing glomerular filtration rate. Furthermore, higher intratubular pressure promotes the leakage of filtrate into the interstitial space and peritubular capillaries and lowers the excretion of the filtered products^4^.

Renal vasculature and glomerular filtration function are also affected by AMGs through stimulation of mesangial cell contraction, proliferation, and apoptosis. These processes are mediated by the elevation of cytosolic calcium, oxidative stress, inflammation, and vasoconstrictor release. Moreover, reduced blood flow and glomerular filtration rate may contribute to aggravate AMG-induced tubular damage by probably limiting the availability of oxygen and nutrients, and at the same time, facilitating oxidative stress, similar to ischemic AKI^4^.

AMG-induced AKI is fatal in some cases. However, patients can survive due to the regenerative capability of tubular cells, which allows them to recover as soon as AMG treatment is stopped^6^. However, even though AKI may be reversible, some patients do not completely recover and usually develop a chronic kidney disease^7^.

Up to now, there is no effective therapeutic alternative to prevent AMG renal toxicity in clinical practice and current treatments are not able to prevent the progression of AKI towards chronic kidney disease. In this context, it is necessary to search for new alternatives contributing to accelerate the regeneration of renal tubular cells and prevent AKI.

Diverse natural and synthetic substances have displayed beneficial actions on AKI induced by AMGs. Antioxidant agents are among the best candidates due to their efficacy in preventing the generation of reactive oxygen species^8^^-^ 10. According to this approach, extracts obtained from plants belonging to Arecaceae family, such as Cocos nucifera and Phoenix dactylifera, have prevented gentamicin-induced AKI in rats^11^^,^ 12.

D-005, a saponified lipid extract obtained from the fruit of *Acrocomia crispa*, endemic palm of Cuba of the Arecaceae family, contains a reproducible mixture of fatty acids, mainly oleic, palmitic, lauric, and myristic while palmitoleic, caprylic, capric, and stearic acids are found in lower concentrations^13^^,^ 14.

Recently, D-005 has shown to prevent pathological changes induced by kidney ischemia/reperfusion in rats, by diminishing serum concentrations of creatinine, uric acid, and urea and lessening tubular histopathological changes in kidney cortex. This was linked to D-005 antioxidant effects, since it decreased the concentrations of malondialdehyde (MDA) and sulfhydryl (SH) groups in plasma and kidney homogenate, as well as it has restored catalase (CAT) enzymatic activity in the kidney^15^.

In addition, previous studies revealed that D-005 exerts anti-inflammatory effects in vitro and in vivo by inhibiting the enzymes 5-lipooxygenase (5-LOX) and cyclooxygenase type 2 (COX-2) and preventing inflammatory infiltration during acute lung damage in mice^16^^,^ 17.

Taking into account the crucial role played by oxidative stress and inflammation in the pathophysiology of AMG-induced AKI, as well as the previous antioxidant and anti-inflammatory effects of D-005, this work aimed to evaluate the effects of D-005 on kanamycin-induced AKI.

## Materials and Methods

### Preparation of extracts

D-005 was obtained from previously authenticated *Acrocomia crispa* ripe fruits (No. 1982-1031, National Botanical Garden, Havana, Cuba) at Laboratory of Pharmaceutical Chemistry, Natural Products Center (CPN) belonging to the National Center for Scientific Research (CNIC), Havana, Cuba. In brief, the fruits were air-dried, milled at 2.36 mm, and extracted with n-hexane at room temperature. Then, solvent was removed at 60 °C under vacuum, in a rotary evaporator, and the resulting oil was saponified at reflux with a hydro alcoholic potassium hydroxide solution (0.5 mol/L). Next, the fatty acids were released with HCl (10 %) until pH 1-2, and the organic phase was washed with water and dried under vacuum at 110 °C^13^^,^ 14.

D-005 batch thus obtained (Batch S-291215) was characterized by gas chromatography, being its fatty acid composition: lauric (41.77 %), oleic (28.10 %), myristic (12.57 %), palmitic (7.83 %), stearic (2.93 %), capric (3.33 %), caprylic (1.73 %), and palmitoleic (0.05%) acids, for a total content of fatty acids of 98.30 %. D-005 was suspended in Tween 65/H_2_O (2 %) just before using.

Grape seed extract (GSE) was used as a reference substance (95 % proanthocyanidin), Batch: R3992407, from Blackmores (Sydney, Australia). GSE was dissolved in acacia gum/H_2_O (1 %) prior to rat administration.

### Animals and experimental protocol

Male Wistar rats (250-300 g) from the National Center for the Production of Laboratory Animals (CENPALAB, Havana, Cuba) were used. Rats were acclimated for 7 days to controlled laboratory conditions (25 ± 2 °C temperature, 60 ± 5 % relative humidity, and 12 hours light/dark cycle) and free access to food and water (standard feed for rodents from CENPALAB). After this period, they were classified as suitable for experimental use. The handling of the animals was carried out according to the Cuban Guidelines for Animal Handling and the Cuban Code of Good Laboratory Practices. The independent ethical board of CPN approved the animal use and study protocols (approval number 007/2018 signed on November 29, 2018).

Rats were randomized into 7 groups (10 rats/group): a negative control (treated with vehicle, Tween 65/H_2_O) and six groups which were administered kanamycin to induce AKI: a positive control group treated with Tween 65/H_2_O, four groups treated with D-005 (25, 100, 200, and 400 mg/kg) and one group treated with GSE (200 mg/kg). The dosage schedule of D-005 was based on its antioxidant and anti-inflammatory previous effects^15^. The chosen dose of GSE has shown antioxidant effects in previous studies and it falls within the range of doses previously used in nephrotoxicity models^18^^-^ 20. D-005, the vehicle, and GSE were administered orally by intragastric gavage in order to guarantee that rats receive the exact dose and according to previous studies^15^^,^ 21. All treatments were administered once daily for seven days.

AKI was induced by daily injections with kanamycin (kanamycin sulfate, 500 mg/kg, i.p.) during seven days concurrently with the course of D-005, vehicle, and GSE treatments, 1 h after receiving the treatments with D-005, vehicle, and GSE. Twenty-four hours after the last treatment (day 8^th^, rats were anesthetized using sodium thiopental (30 mg/kg) and blood samples were taken from the abdominal aorta for blood biochemistry (serum concentrations of uric acid and urea) and determination of oxidation parameters (concentrations of MDA and SH groups as well as CAT activity). Immediately, both kidneys were removed; the left one was used for histopathological analysis and the right one was used for the determination of the oxidation parameters. Kidneys used for biochemical analysis were homogenized using a blade homogenizer (Ultra-Turrax) in an ice bath and a suitable buffered solution according to the technique to be performed. The homogenized samples were stored at -20 °C.

### Blood biochemistry determinations

Uric acid and urea concentrations were measured in serum samples using Spinreact Signature reagent kits in an UV-Genesys 10S Spectrophotometer. The uric acid readings were made at 520 nm and reported as µmol/L, whereas urea readings were made at 340 nm and reported as mg/dL.

### Histopathological processing and analysis

Kidneys were sectioned through the hilum following the sagittal plane and immediately immersed in 4 % buffered formalin. Subsequently, samples were subjected to ethanol, xylol and paraffin passes. Then, they were embedded in paraffin blocks and 3 µm-thick sections were obtained using a Sakura microtome. Two histological slides per animal were prepared: one slide was stained with hematoxylin and eosin, and the second one with periodic Schiff's acid (PAS) 22.

Histopathological analysis was performed from the upper to the lower pole of each kidney hemi-section. Morphology of glomeruli and proximal tubules, as well as the condition of the interstitium, were studied by means of a Zeiss Primo Star light microscope. The following variables were assessed:

- Glomerular damage: evaluated using the following 0-2 scores: 0: normal appearance; 1: mesangial expansion (wide, strongly stained areas in the mesangium) and/or mesangial hypercellularity (presence of more than three nuclei in the mesangium); 2: glomerular tuft retraction with increased Bowman's space.
- Tubular damage: assessed using the following 0-4 scores: 0: normal structure; 1: some tubules with damaged or necrotic cells; 2: isolated areas of tubular necrosis; 3: frequent areas of tubular necrosis; 4: widespread or diffuse necrosis.
- Interstitial damage: evaluate by the presence or absence of inflammatory cells in the interstitium using the following scores (0-2): 0: absence of inflammatory cells; 1: scarce inflammatory cells (1-5 cells per field); 2: abundant inflammatory cells (more than 5 cells/field).
- Global renal damage: calculated from the sum of the values of glomerular damage, tubular damage, and interstitial damage assigned to each animal.

### Determination of the mda concentration

The determination of thiobarbituric acid reactive substances (TBARS) was carried out according to a previous described technique^23^. Briefly, the reaction mixture (plasma or kidney homogenate) was treated with 0.2 mL sodium dodecyl sulfate (SDS) (8.1 %), 1.5 mL of acetic acid (20 %, pH 3.5), and 1.5 mL of an aqueous solution of thiobarbituric acid (0.8 %) and then heated at 95 °C. In order to avoid the production of additional peroxidation that could constitute an error in the determination during the heating process, butylated hydroxytoluene (1 mmol/L) was added to the medium. Samples were cooled and 5 mL of an n-butanol-pyridine mixture (15:1 v/v) was added to each one. They were then vigorously shaken using a vortex, centrifuged at 4,000 rpm for 20 min. The organic layer was removed and its absorbance was read at 534 nm in a spectrophotometer. For calculations of TBARS levels, a standard curve with MDA bis(dimethyl acetal) was plotted. MDA values were reported as nmol MDA/mg of protein. Protein concentrations were obtained by the modified Lowry method^24^.

### Determination of sh group concentration

In brief, 600 µL tris-ethylenediamine tetraacetic acid buffer (20 mmol/L), pH 8.2, 40 µL 5,5-dithiobis-(2-nitrobenzoic acid) (DTNB) (10 mmol/L), and 3.16 mL ethanol (100 %) were added to an aliquot of 200 µL of the sample (plasma or kidney homogenate). The mixture was incubated during 15-20 min at room temperature and subsequently centrifuged at 3,000 rpm for 10 min. The supernatant absorbance was read at 412 nm. A blank was prepared with DTNB, and total concentration of SH was calculated using 13,600 cm-1M-1 absorptivity and reported in mmol/L^25^.

### Assessments of cat activity

Firstly, H_2_O_2_ removal was controlled at 240 nm for 5 min in a spectrophotometer. To 10 µL of sample (plasma or kidney homogenate), 2.89 mL potassium phosphate buffer (50 mmol/L, pH 7.4) was added. The reaction started by adding 0.1 mL H_2_O_2_ to a final volume of 3 mL at 25 °C. CAT activity was calculated by the molar extinction coefficient (43.6 × 10^-3^ and was expressed in IU/min mg of protein × 10^-1^ 26.

The inhibition percentages of D-005 and GSE groups for the variables uric acid, urea, global kidney damage, MDA, and SH groups were calculated using the following formula:


$$I=100-\frac{\left(T-\mathrm{NC}\right)\;x\;100}{\mathrm{PC}-\mathrm{NC}}$$

wherein: I is inhibition, T is treated group mean, NC is Negative control mean, and PC is Positive control mean.

For CAT activity, the restoration percentages of D-005 and GSE groups were calculated using the following formula:


$$R=100-\frac{\left(\mathrm{NC}-T\right)\;x\;100}{\mathrm{NC}-\mathrm{PC}}$$

wherein: R is restoration, T is treated group mean, NC is Negative control mean, PC: Positive control mean.

### Statistical analysis

Comparisons between groups was performed using the non-parametric Mann Whitney U test. Differences were considered statistically significant at a value of p<0.05. Statistical analyses were performed using the Prism 5.0 software for Windows (Graphpad Software Inc., San Diego, CA, USA). Dose/effect relationship analyses were based on the linear regression and correlation method using Origin software version 5.0, Microcal Software, Inc. 1991-1997.

## Results

### Effects of D-005 on aki biomarkers

Kanamycin caused a marked and significant increase on uric acid and urea serum levels in the positive control group compared to the negative control group. GSE, used as a reference substance, markedly and significantly decreased the concentrations of uric acid and urea, compared to the positive control, reaching 93.0 and 64.3 % inhibition, respectively (Table 1).

**Table 1:** Effects of D-005 on serum creatinine and uric acid concentrations in rats with kanamycin-induced acute kidney injury

Treatment	Uric acid (µmol/L )	I (%)	Urea (mg/dL)	I (%)
Negative control (vehicle)	66.23 ± 7.17 ***	-	81.31 ± 4.08 ***	-
Positive control (vehicle + kanamycin)	119.48 ± 8.49	-	192.90 ± 22.47	-
D-005 (25 mg/kg) + kanamycin	104.52 ± 11.2	28.1	170.11± 22.96	20.4
D-005 (100 mg/kg) + kanamycin	83.35 ± 8.51 *	67.8	129.20 ± 13.24 *	57.1
D-005 (200 mg/kg) + kanamycin	69.14 ± 7.11 ***	94.5	116.01 ± 10.12 **	68.9
D-005 (400 mg/kg) + kanamycin	68.06 ± 6.51 ***	96.6	114.45 ± 9.66 **	70.3
GSE (200 mg/kg) + kanamycin	69.95 ± 4.98 ***	93.0	121.13 ± 14.37 *	64.3

GSE: grape seed extract, I: inhibition. Data are reported as mean ± SEM (standard error of the mean).

* p<0.05;

** p<0.01;

*** p<0.001; Comparison with the positive control group. Mann Whitney-U test.

D-005 significantly diminished in a dose-dependent way the increase in uric acid (p = 0.023, r = 0.976) and urea (p = 0.039, r = 0.960) serum levels, starting from the dose of 100 mg/kg and reaching the highest percentages of inhibition at 400 mg/kg (96.6 % in uric acid and 70.3 % in urea) (Table 1).

### Effects of D-005 on renal histopathology

**Figure 1:**
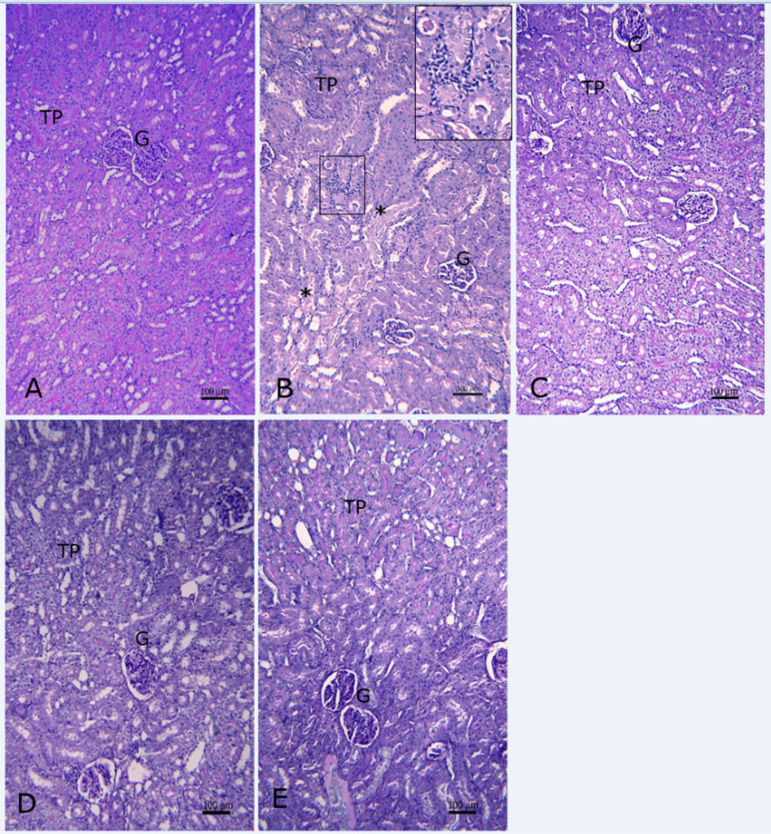
Renal cortex of rats with PAS staining. A representative image of each experimental group is shown. A) Negative control, B) Positive control (Kanamycin), C) Grape seed extract (200 mg/kg) + Kanamycin, D) D-005 25 mg/kg + Kanamycin, E) D-005 400 mg/kg + Kanamycin. Bars- 100 µm. G: glomeruli; TP: proximal tubules; *tubular necrosis; rectangle in B encloses peritubular inflammatory infiltrate (insert at higher magnification in the upper right corner).

The normal structure of the kidney parenchyma was observed in the rats of the negative control group, showing prominent PAS-positive brush borders in proximal tubules (Figure 1A). Rats belonging to the positive control group presented an increased glomerular mesangium, mesangial hypercellularity, and on occasions, retraction of glomerular tuft with enlarged Bowman's space, loss of brush borders and frequent tubular necrosis, as well as peritubular inflammatory infiltrate in the middle zone of kidney cortex (Figure 1B).

Additionally, the positive control group displayed marked and significant increase in histopathological scores of glomerular, tubular, and interstitial damage, as well as global kidney damage, which was obtained by the contribution of these three variables (Tables 2 and 3).

**Table 2:** Effects of D-005 on renal histopathological scores of glomerular, tubular and interstitial damages in rats with kanamycin-induced acute kidney injury

Treatment	Glomerular damage (score)	I (%)	tubular damage (score)	I (%)	Interstitialdamage (score)	I (%)
Negative control (vehicle)	0.80 ± 0.13 ***	-	0.10 ± 0.10 ***	-	0.0 ± 0.0 ***	-
Positive control (vehicle + kanamycin)	1.90 ± 0.10	-	2.84 ± 0.23	-	1.90 ± 0.10	-
D-005 (25 mg/kg) + kanamycin	1.24 ± 0.25 *	59.6	1.80 ± 0.29 *	37.9	1.40 ± 0.16 *	26.3
D-005 (100 mg/kg) + kanamycin	1.22 ± 0.25 *	61.4	1.78 ± 0.33 *	38.7	1.20 ± 0.29 *	36.8
D-005 (200 mg/kg) + kanamycin	1.12 ± 0.18 **	70.6	1.71 ± 0.25**	41.2	1.13 ± 0.18 **	40.5
D-005 (400 mg/kg) + kanamycin	1.10 ± 0.10 ***	72.5	1.64 ± 0.12***	43.8	1.10 ± 0.10 ***	42.1
GSE (200 mg/kg) + kanamycin	1.10 ± 0.23 **	72.5	1.60 ± 0.34 *	43.9	1.40 ± 0.16*	26.3

GSE: grape seed extract; I: inhibition. Data are reported as mean ± SEM (standard error of the mean).

* *p<0.05*;

** *p<0.01;*

*** *p<0.001*; Comparison with the positive control group. Mann Whitney U test.

**Table 3:** Effects of D-005 on global renal damage in rats with kanamycin-induced acute kidney injury

Treatment	Global renal damage (score)	I (%)
Negative control (vehicle)	0.90 ± 0.10 ***	-
Positive control (vehicle + kanamycin)	6.54 ± 0.36	-
D-005 (25 mg/kg) + kanamycin	4.88 ± 0.38 *	29.4
D-005 (100 mg/kg) + kanamycin	4.01 ± 0.63 *	44.9
D-005 (200 mg/kg) + kanamycin	3.96 ± 0.35 **	45.7
D-005 (400 mg/kg) + kanamycin	3.92 ± 0.14***	48.2
GSE (200 mg/kg) + kanamycin	4.2 ± 0.35 **	41.5

GSE: grape seed extract; I: inhibition. Data are reported as mean ± SEM (standard error of the mean).

* *p<0.05*;

** *p<0.01*;

*** *p<0.001*; Comparison with the positive control group. Mann Whitney U test.

GSE, the reference substance in this study, partially prevented kanamycin-induced histopathological changes in kidney structures, showing numerous proximal tubules with well-preserved brush borders (Figure 1C). GSE was also found to significantly prevent increased glomerular, tubular, and interstitial damage scores, as well as kanamycin-induced global kidney damage (Tables 2 and 3).

Treatments with D-005, from the lowest dose evaluated, protected kidney parenchyma structures (glomeruli, proximal tubules, and interstitium) from the injury induced by kanamycin (Figure 1D, E) and caused a significant decrease of the histopathological variables: glomerular, tubular, and interstitial damage, as well as global kidney damage. The effects of D-005 over interstitial and global kidney damages were dose-dependent (p = 0.020, r = 0.980 and p = 0.057, r = 0.942, respectively). The highest percentages of inhibition were obtained with the dose of 400 mg/kg of D-005 in both variables (Tables 2 and 3).

### Effects of d-005 on oxidative stress markers

Rats in the positive control group, treated with kanamycin, achieved significantly higher values of MDA and SH groups, as well as a depletion of CAT activity compared to the negative control, both in plasma and kidney homogenate (Tables 4 and 5).

**Table 4:** Effects of D-005 on plasma MDA and SH group concentrations and CAT activity in rats with kanamycin-induced acute kidney injury

Treatment	MDA (nmol/mg of pt)	I (%)	Grupos SH (mmol/L)	I (%)	CAT (UI/min/mg of pt)	R (%)
Negative control (vehicle)	1.41 ± 0.13 ***	-	0.49 ± 0.02 ***	-	4.14 ± 0.40 ***	-
Positive control (vehicle + kanamycin)	4.50 ± 0.24	-	0.80 ± 0.06	-	1.02 ± 0.19	-
D-005 (25 mg/kg) + kanamycin	2.99 ± 0.18 ***	48.7	0.63 ± 0.04 *	53.4	3.27 ± 0.24 ***	72.2
D-005 (100 mg/kg) + kanamycin	2.85 ± 0.22 ***	53.5	0.56 ± 0.03 **	75.9	3.52 ± 0.35 ***	80.2
D-005 (200 mg/kg) + kanamycin	2.38 ± 0.31 ***	68.5	0.54 ± 0.04 **	82.3	3.97 ± 0.18 ***	94.7
D-005 (400 mg/kg) + kanamycin	2.14 ± 0.29 ***	76.5	0.52 ± 0.03 **	88.5	4.04 ± 0.26 ***	97.0
GSE (200 mg/kg) + kanamycin	2.22 ± 0.28 ***	73.7	0.60 ± 0.02 **	64.8	3.53 ± 0.26 ***	80.7

GSE: grape seed extract; I: inhibition; R: restoration; pt: protein. Data are reported as mean ± SEM (standard error of the mean).

* *p<0.05*;

** *p<0.01*;

*** *p<0.001*; Comparison with the positive control group. Mann Whitney U test.

**Table 5:** Effects of D-005 on MDA and SH groups concentrations and CAT activity in kidney homogenate in rats with kanamycin-induced acute kidney injury injury

Treatment	MDA (nmol/mg de pt)	I (%)	Grupos SH (mmol/L)	I (%)	CAT (UI/min/mg de pt)	R (%)
Negative control (vehicle)	1.98 ± 0.40 ***	-	0.67 ± 0.02 ***	-	2.13 ± 0.20 ***	-
Positive control (vehicle + kanamycin)	5.69 ± 0.21	-	1.14 ± 0.06	-	0.55 ± 0.06	-
D-005 (25 mg/kg) + kanamycin	2.58 ± 0.4 ***	83.7	0.89 ± 0.04 **	52.8	1.03 ± 0.10 **	30.5
D-005 (100 mg/kg) + kanamycin	2.02 ± 0.23 ***	98.8	0.74 ± 0.04 ***	85.5	1.42 ± 0.22 **	55.4
D-005 (200 mg/kg) + kanamycin	1.93 ± 0.21 ***	100	0.71 ± 0.06 ***	90.9	1.60 ± 0.24 ***	66.4
D-005 (400 mg/kg) + kanamycin	1.93 ± 0.30 ***	100	0.67 ± 0.05 ***	99.0	1.65 ± 0.45 ***	69.6
GSE (200 mg/kg) + kanamycin	2.42 ± 0.51 ***	88.1	0.72 ± 0.04 ***	90.1	1.40 ± 0.30 **	53.4

GSE: grape seed extract; I: inhibition; R: restoration; pt: protein. Data are reported as mean ± SEM (standard error of the mean).

* *p<0.05*;

** *p<0.01*;

*** *p<0.001*; Comparison with the positive control group. Mann Whitney U Test.

The reference substance (GSE) significantly decreased MDA and SH group concentrations and partially restored CAT activity in plasma and kidney homogenate (Tables 4 and 5).

D-005 at all the tested doses (25, 100, 200, and 400 mg/kg) significantly diminished the increase in MDA concentration in both plasma and kidney homogenate, reaching highest percentages of inhibition at doses of 400 mg/kg in plasma and 200 mg/kg in tissue sample (76.5 and 100 % respectively) (Tables 4 and 5).

In addition, D-005 significantly decreased SH groups in a dose-dependent manner both in plasma (p = 0.012, r = 0.987) and kidney homogenate (p = 0.025, r = 0.975), being the highest percentage of inhibition at the dose of 400 mg/kg (88.5 % in plasma and 99.01 % in kidney homogenate) (Tables 4 and 5).

The effects of D-005 (25-400 mg/kg) on CAT activity in plasma and kidney homogenate are also shown in Tables 4 and 5. D-005, from the lowest dose tested, restored CAT activity in a significant and dose-dependent manner (p = 0.039, r = 0.960 in plasma and p = 0.019, r = 0.981 in kidney homogenate), reaching the highest recovery percentage at the dose of 400 mg/kg (97.02 % in plasma and 69.6 % in kidney homogenate).

## Discussion

The results of this study demonstrate the protective effect of repeated oral doses of D-005 on kanamycin-induced AKI.

The biochemical and histopathological changes in the positive control group (kanamycin-treated rats) is in accordance to those described by other authors under similar conditions^27^^,^ 28. The increase in serum concentrations of uric acid and urea in this group indicates a decrease in the glomerular filtration rate, which constitutes a hallmark of AKI^29^^-^ 31, being the increase of urea in the Positive control group in agreement with values reported in diverse animal models of aminoglycoside-induced AKI^8^.

Moreover, although the serum creatinine-concentration quantification constitutes the gold standard in the clinical diagnosis of AKI, in the model of aminoglycoside-induced nephrotoxicity diagnosis is better established by the increase in urea concentration -sometimes expressed as BUN - since urea increases 60 times more than creatinine in this type of experimental damage^8^^,^ 10. Therefore, not having quantified the creatinine concentration cannot be considered a limitation of this study.

Regarding our histopathological findings, the nephrotoxic treatment impaired the morphology of renal cortex parenchyma, which showed glomerular alterations, tubular necrosis, and inflammatory infiltrate close to necrotic tubules. These results resemble those of previous outcomes based on gentamicin nephrotoxicity models, where the death of tubular cells has been recognized as the primary event due to the entry of AMG therein, whereas the infiltration of inflammatory cells and glomerular changes are considered secondary events promoted by tubular dysfunction^32^^,^ 33.

As expected, GSE, used as a reference substance, markedly and significantly prevented the rise of uric acid and urea serum concentrations induced by kanamycin. Likewise, GSE significantly decreased the histopathological damage of kidney structures, compared to the positive control group, in agreement with previous reports^34^.

Taking into account the increase in biochemical parameters and histopathological alterations of kidney structures found in the positive control group, which are both typical of AKI, and that GSE, used as a reference substance, prevented the changes induced by kanamycin, the rat model used and the results obtained in our experimental conditions are validated.

In the present work, it was shown that D-005 partially restored glomerular filtration rate, since it decreased serum concentrations of uric acid and urea. The minimum effective dose of D-005 in both parameters was 100 mg/kg. The protection of renal function was accompanied by the preservation of renal parenchyma structures observed in the histopathological study, although the morphological protection was evidenced from the minimum D-005 dose (25 mg/kg).

Nephrotoxic treatment with gentamicin has been shown to cause an increase in COX-2 in rats and mice, which can be reversed after administration of anti-inflammatory agents such as hesperidin, alpha linolenic acid, and an extract of Hypericum perforatum^35^^-^ 37. Further studies will elucidate if the anti-inflammatory effect of D-005, histopathologically proven in this study by decreasing the infiltration of inflammatory cells in the renal parenchyma is associated, at least partially, to its dual inhibitory capacity on 5- LOX and COX-2 enzymes, similar to flavocoxid^16^^,^ 38.

The renal accumulation of AMGs, like kanamycin, causes direct toxic effects on tubular cells, accompanied by microvascular inflammation and ischemia, which promote ROS generation. Furthermore, the biotransformation of these drugs in the kidney is carried out by kidney enzymes such as cytochrome P-450, which also induces the generation of ROS. Accordingly, AMGs induce lipid peroxidation and renal dysfunction mediated by increased ROS, as well as iron release from the mitochondria of cortical tubular cells^39^. This explains the results obtained in the present study in terms of increased concentrations of MDA and SH groups, as well as depleted CAT activity in both plasma and kidney homogenate in the positive control group.

GSE protected from kanamycin-induced oxidative damage by decreasing MDA and SH group concentrations and partially restoring CAT activity, both in plasma and kidney homogenate. The nephroprotective effects of GSE have been proven before in models of nephrotoxicity induced by other AMGs, such as amikacin and gentamicin, in relation to its anti-apoptotic and antioxidant properties^34^^,^ 40. The usefulness of GSE as a reference substance is proved herein using the model of kanamycin-induced AKI.

Antioxidants are the most studied protective agents in animal models of nephrotoxicity and have been shown to be the most valuable preventive strategy against AMG-induced AKI. In this sense, the best protection results have been obtained with antioxidant extracts from plants, being administered concomitantly with the AMG, either simultaneously or as a pre-treatment on the same day^10^. Examples of this are the antioxidant extracts from two species of Arecaceae (Cocos nucifera and Phoenix dactylifera) exhibiting protective effects against AKI, which were administered concomitantly with gentamicin^11^^,^ 12.

Therefore, the protective effects of D-005 on AKI verified in this study could also be attributed, at least partially, to its antioxidant properties, which is supported by the decrease of lipid peroxidation and protein oxidation, showed with reduction of MDA and SH groups respectively, and the enhancement of endogenous antioxidants, by restoring CAT activity.

The antioxidant and anti-inflammatory effects of D-005 could be attributed to its content of fatty acids as lauric acid, oleic acid, and myristic acid, which have shown these effects previously^41^^,^ 42. In addition, myristic acid has revealed a nephroprotective effect by reducing tubular necrosis in an experimental model of AKI^43^. Finally, the fact that extracts obtained from Cocos nucifera and Phoenix dactylifera, palms with fatty acid compositions similar to D-005, have demonstrated protection against gentamicin-induced nephrotoxicity supports our hypothesis^11^^,^ 12.

In this context, this study constitutes the first evidence of the nephroprotective efficacy of this mixture of fatty acids obtained from Cuban *Acrocomia crispa* on aminoglycosides-induced renal damage. Moreover, it was in accordance with previous results in preventing ischemia-reperfusion-induced renal damage^15^, and further studies will clarify the mechanisms by which D-005 exerts its nephroprotective effect in both models of AKI.

Overall, we can suggest that D-005 is a new substance with considerable potential benefit on AKI, either as an antioxidant supplement or as a treatment for this pathology.

In conclusion, the administration of repeated oral doses of D-005, concomitant with kanamycin, significantly protected rats from AKI, which could be related to the antioxidant and anti-inflammatory actions of this plant extract.
